# A Hill type equation can predict target gene expression driven by p53 pulsing

**DOI:** 10.1002/2211-5463.13179

**Published:** 2021-05-27

**Authors:** Xiaomin Shi

**Affiliations:** ^1^ Department of Mathematics and International Center for Quantum and Molecular Structures Shanghai University China

**Keywords:** fold change, hill equation, mRNA dynamical model, p53 pulse, target gene expression patterns

## Abstract

Many factors determine target gene expression dynamics under p53 pulsing. In this study, I sought to determine the mechanism by which duration, frequency, binding affinity and maximal transcription rate affect the expression dynamics of target genes. Using an analytical method to solve a simple model, I found that the fold change of target gene expression increases relative to the number of p53 pulses, and the optimal frequency, 0.18 h^−1^, from two real p53 pulses drives the maximal fold change with a decay rate of 0.18 h^−1^. Moreover, p53 pulses may also lead to a higher fold change than sustained p53. Finally, I discovered that a Hill‐type equation, including these effect factors, can characterise target gene expression. The average error between the theoretical predictions and experiments was 23%. Collectively, this equation advances the understanding of transcription factor dynamics, where duration and frequency play a significant role in the fine regulation of target gene expression with higher binding affinity.

## Graphical abstract

I discovered a Hill‐type equation that can characterise target gene expression driven by p53 pulsing. The average error between the theoretical predictions and experiments was 23%. Altogether, this equation advances the understanding of transcription factor dynamics, where duration and frequency play a significant role in the fine regulation of target gene expression with higher binding affinity.

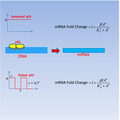

Half of all human cancers contain p53 gene mutations. Further, in many other cancers, the function of the p53 protein is eliminated. Drugs targeting the p53 pathway have been developed for these two cancer types [1, 2, 3]. Information on environmental stimuli is encoded by signalling dynamics [4]. Dynamic information, such as duration, amplitude, decay rate, and signal rise time, can overcome extrinsic noise, thereby enhancing information transfer and increasing the accuracy of biochemical signalling networks [5, 6]. Cancer mutations and drugs can disturb target signalling molecule dynamics, resulting in different cell decisions, including proliferation, cell cycle arrest and senescence [7, 8, 9].

The transcription factor, p53, connects cellular signal transduction networks to the transcription network. In response to gamma radiation, p53 temporal behaviours in individual cells express repeated pulses with fixed amplitude, duration and frequency, and the number of pulses increases with an increase in radiation dose [10]. In response to UV radiation, p53 dynamics appear as a single prolonged pulse, whose amplitude and duration depend on the dose of stimulation [11]. By pharmacological interference, changing p53 dynamics from pulsed to sustained can directly drive senescence [8]. Cells use pulsed and sustained time patterns to encode different stimuli; however, the decoding mechanism is not fully understood.

Cells discriminate between pulsed and sustained signalling by an accurate biochemical mechanism to determine the cell outcome. The dynamics of p53 target gene expression depend on pulsed signalling. p53 signalling can be decoded by mRNA dynamics. p53 pulses drive diverse gene expression dynamics. The mRNA decay rate with the frequency of the p53 pulse determines this diversity [9, 12]. The role of p53 signalling has also been investigated using p53 DNA‐binding dynamics [12, 13, 14].

In response to ionising radiation, observations from cell populations have shown that DNA‐binding dynamics are correlated with the affinity of p53 binding [14]. The level of p53 binding to the *CDKN1A* (p21) or *MDM2* promoter with higher affinity is increased significantly; however, the level of p53 binding to the *PIG3* promoter with lower affinity is minor, which results in a lower PIG3 mRNA level [14]. Higher binding affinity results in a higher mRNA induction [15]. The diversity of p53 DNA‐binding dynamics caused by different affinities is consistent with theoretical predictions [13].

Different maximal values of p53 DNA‐binding pulses are observed under two full p53 input pulses [12]. Evidently, the difference between the DNA‐binding level only results from the different promoter affinities. In addition, the maximal CDKN1A mRNA fold change was found to be more than 11‐fold that of BAX [12]. Considering that the decay of CDKN1A mRNA is much faster than that of BAX [9], and the affinity of CDKN1A is markedly higher than that of BAX [16], the higher CDKN1A mRNA level is not only correlated with the decay rate, but also correlated with the binding affinity. Under the same p53 input pulses, the observations show that the maximal expression level in single cells is ranked by binding affinity: CDKN1A and GADD45A with higher affinity, MDM2 with medium affinity and BAX with lower affinity [8, 16]. The dynamic pattern of CDKN1A mRNA in the cell population [12, 14]corresponds to weak pulsing [9].

The ligand pulsing effect is not included in the Hill equation. However, we discovered a modified Hill equation for the average p53 DNA‐binding probability [13]:

$${\bar{P}}_{\text{pulsed}}=\frac{{\bar{\left[S\right]}}^{n}}{\gamma^{n‐1}K_{A}^{n}+{\bar{\left[S\right]}}^{n}},\bar{\left[S\right]}=\gamma A,$$

where $\bar{\left[S\right]}$ is the average concentration of the pulsed signalling molecule, $K_{A}$ is the dissociation constant, γ is the duty cycle defined as the ratio of the pulsing duration to the period, and *A* is the amplitude of the square wave input. The duty cycle can be measured for some pulsed transcription factors [17]. For sustained signalling γ = 1, the classic Hill equation is reduced. This equation demonstrates that pulsed signalling enhances the roles of receptors with high binding affinity by reducing the average signal molecule concentration required for activation. As a result, p53 pulsing increases the sensitivity of DNA‐binding dynamics to lower p53 levels. However, this remains to be experimentally confirmed. Interestingly, we wanted to determine whether there was a similar equation to govern target gene expression upon p53 pulsing.

Compared to sustained signalling, pulsing has other advantages. In fact, pulsing enables diverse cellular functions [18]. Using a simple dynamical model of gene promoter in response to pulsed and sustained transcription factor signalling, a pulsing signal can produce a more constant protein level than a sustained signal and reduce the noise in gene expression [19].

As p53 pulse frequency increases by treating cells with small molecular Nutlin‐3, which binds to the MDM2, recent studies on p53 target gene expression dynamics have examined the role of mRNA decay rate and p53 pulse frequency [9, 12]. However, the detailed mechanism by which duration or duty cycle, binding affinities and the maximal transcription rate together with mRNA decay rate affect the expression dynamics remains unclear. Using a simple mRNA dynamical model, we investigated the effect of the duty cycle, binding affinity, maximal transcription rate and mRNA decay rate on mRNA dynamics.

Some decoding models of pulsed molecular signalling dynamics can be analytically solved [13, 20, 21, 22]. The average of p53 dynamics over cell populations expressed damped oscillations. Assuming that p53 dynamics can be modelled by the sum of a constant and a sinusoidal term, an analytical solution for target gene expression was obtained; however, this solution could not include binding affinity and duration or duty cycle [9]. In single cells, p53 dynamics show digital pulses. In this case, an analytical solution has not been obtained. Assuming that the signalling dynamics are simplified to a piecewise function, the duration or duty cycle can be introduced into the model, a full analytical solution is easily obtained and simplified asymptotically, and the nature of pulsed signalling is revealed [13, 22]. Here, we attempted to find analytical solutions under pulsed and sustained p53 signalling to clearly indicate the dependence of mRNA dynamics on multiple parameters; this is an essential step in understanding the decoding principle of p53 signalling.

## Methods

### Mathematical model of p53 target gene expression dynamics and its analytical solution

There are two types of p53 target gene expression models: with [23] or without time delay [24, 25]. In the deterministic model, the time delay makes the oscillations robust to parameter changes [26]. Some models describe the stochastic effects of gene expression [27, 28, 29]. Under certain conditions, the predictive results of the deterministic models are similar to those of stochastic simulations [29]. To understand p53 target gene expression dynamics, we developed an ordinary differential equation system.

(1) $$\frac{dP(t)}{\mathrm{dt}}=\left(1‐P(t)\right)k_{1}{\left[S(t)\right]}^{n}‐k_{2}P\left(t\right),$$

(2) $$\frac{d\text{mRNA}(t)}{\mathrm{dt}}=\beta_{0}^{\prime }+\beta^{\prime }P\left(t\right)‐\alpha \text{mRNA}\left(t\right),$$

where

$$\begin{array}{c} \left[S(t)\right]=\left\{\begin{array}{cc} A, & (i‐1)T\leq t<(i‐1)T+\Delta \\ 0, & (i‐1)T+\Delta \leq t<iT \end{array}\right.\;i=1,2,\ldots ,\;\text{for pulsed signaling},\;\\ \left[S(t)\right]=\begin{array}{cc} \;A, & 0\leq t\leq iT, \end{array}\;i=1,2,\ldots ,\;\text{for sustained signaling}. \end{array}$$

Equation (1) describes the p53 DNA‐binding dynamics [13]. Under constant signalling, its steady state yields the classical Hill equation, which is initially just empirical curve fitting [30, 31]. Later, it was found that the Hill equation can be obtained from the reactions in which *n* molecules of the ligand bind cooperatively to a receptor, if the intermediary reactions are very fast [32]. Therefore, the Hill equation is just the steady state of such binding dynamics, as detailed intermediate steps are omitted. Although the Hill coefficients *n* are integers, in practice, *n* may be a noninteger to derive the best fit [32].

Equation (2) represents the mRNA dynamics of the p53 target genes. *P*(*t*) and mRNA(*t*) denote the binding probability and target gene mRNA concentration, respectively, while [*S*(*t*)] is the piecewise constant function of the input p53 dynamics. *T* is the amplitude and Δ is the duration; *k*_1_ and *k*_2_ denote the rate constants of association and disassociation, respectively; $\beta_{0}^{\prime }$ is the basal transcription rate, which is low and activated by general transcription factors; and β^′^ is the maximal transcription rate activated by p53, while α is the mRNA decay rate. As the binding of p53 DNA is faster than gene transcription, under the quasi‐steady‐state assumption that $\alpha /k_{2}\ll 1$, Equation (2) becomes

(3) $$\frac{d\text{mRNA}(t)}{\mathrm{dt}}=\beta_{0}^{\prime }+\beta^{\prime }\frac{{\left[S(t)\right]}^{n}}{K_{A}^{n}+{\left[S(t)\right]}^{n}}‐\alpha \text{mRNA}\left(t\right),$$

where the dissociation constant is defined as $K_{A}={\left(k_{2}/k_{1}\right)}^{1/n}$. If $m_{0}={\beta^{\prime }}_{0}/\alpha$ represents the basal level of mRNA dynamics, then $\text{mRNA}\left(0\right)=m_{0}$. If $m\left(t\right)=\text{mRNA}\left(t\right)/m_{0}$, which is the fold change of mRNA level, Eqn (3) can be written as:

(4) $$\frac{dm(t)}{\mathrm{dt}}=\alpha \left(1+\beta \frac{{\left[S(t)\right]}^{n}}{K_{A}^{n}+{\left[S(t)\right]}^{n}}‐m\left(t\right)\right),$$

where $\beta =\beta^{\prime }/\beta_{0}$ denotes the ratio of the maximal transcription rate to the basal transcription rate, and this is the fold change in maximal transcription. The initial condition is

(5) m(0)=1.

Therefore, the steady state for sustained signalling is

(6) $$m_{\text{st,sus}}=1+m_{d}=1+\frac{\beta A^{n}}{\left(K_{A}^{n}+A^{n}\right)},$$

where *m*_d_ is the increase in steady state from sustained p53 input dynamics, which has no relationship with mRNA lifetime.

By comparing Eqn (4) with that of p53 DNA‐binding dynamics [13], the decay rate α is constant. As a result, it is a reduced equation. Thereafter, by omitting related terms in the solution of reference [13], considering the initial condition (Eqn 5), and inferring that the initial condition of equation in the reference [13] is zero, we can obtain the analytical solutions driven by pulsed signalling:

(7) $$\begin{array}{c} m_{i}\left(\xi_{i}\right)=\begin{array}{cc} 1+m_{d}\left(1‐\frac{e^{‐\alpha \xi_{i}}}{1‐e^{‐\alpha T}}\left(\left(1‐e^{\alpha (\Delta ‐T)}\right)+e^{‐i\alpha T}\left(e^{\alpha \Delta }‐1\right)\right)\right), & 0\leq \xi_{i}<\Delta , \end{array}\;\\ m_{i}\left(\xi_{i}\right)=\begin{array}{cc} 1+m_{d}\frac{\left(e^{\alpha \Delta }‐1\right)\left(1‐e^{‐i\alpha T}\right)}{1‐e^{‐\alpha T}}e^{‐\alpha \xi_{i}}, & \Delta \leq \xi_{i}\leq T \end{array}\;\\ \xi_{i}=t‐\left(i‐1\right)T. \end{array}$$

The solution driven by sustained signalling is

(8) $$m_{\text{sus}}\left(t\right)=1+m_{d}\left(1‐e^{‐\alpha t}\right),$$

By inspecting Eqn (7), α, Δ, and *T* are variables of the exponential function; these parameters can dominantly affect mRNA dynamics.

## Results

### A model can demonstrate that the half‐life of mRNA changes the expression dynamics of p53 target genes

**Fig. 1:**
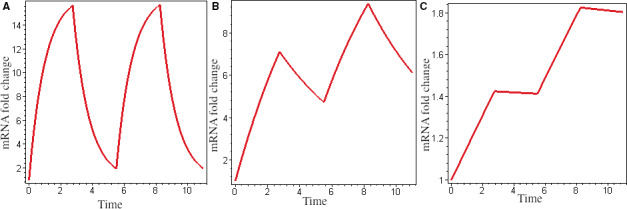
mRNA half‐life changes mRNA dynamics. *K_A_* = 21 nm, A = 60 nm and β = 18. (A) Strongly pulsing (α = 1); (B) weakly pulsing (α = 0.18); and (C) rising dynamics (α = 0.01)

In response to pulsed p53 input dynamics, the mRNA dynamics of p53 target genes were found to be determined by α [9, 12]. Here, we reproduced this situation. If T=5.5h [9], the Hill coefficient n=1.8 [16]: under two p53 input pulses, decreasing α from 1 to 0.18 h^−1^ and 0.1 h^−1^, which correspond to αT>1, αT=1 and αT<1, respectively, strongly pulsing (Fig. 1A) to weak pulsing (Fig. 1B) and rising dynamics (Fig. 1C). The expression dynamics of p53 target genes include these three distinct patterns [9]. Furthermore, the maximal mRNA level was found to increase with a decrease in mRNA lifetime, which is consistent with the experiment [12]. A very high level of mRNA may be harmful to cells; thus, cells chose strongly pulsing to lower the average expression level. Conversely, a sufficiently high level of mRNA is necessary for cells to perform function; therefore, cells use rising dynamics to increase their average expression levels.

### Analytical characterisation of strongly pulsing, weakly pulsing and rising gene expression

From Eqn (7), we can obtain the steady‐state fold change at the end of the pulse:

(9) $$m_{\text{st}}\left(T\right)=\lim_{i\to \infty }m_{i}\left(T\right)=1+m_{d}\frac{e^{\alpha \Delta }‐1}{e^{\alpha T}‐1},$$

For rising dynamics, αT≪1, the following is achieved:

(10) $$m_{\text{st}}\left(T\right)=m_{\text{st,pulsed}}=1+\gamma m_{d}.$$

This is the steady state of rising expression dynamics.

For strongly pulsing, αT≫1,

(11) $$m_{\text{st}}\left(T\right)=1.$$

This is the steady state of strongly pulsing expression.

For weakly pulsing, αT=1,

(12) $$m_{\text{st}}\left(T\right)=1+m_{d}\frac{e^{\gamma }‐1}{e‐1}<1+\gamma m_{d}$$

which belongs to the steady state of weakly pulsing expression.

Therefore, each gene with rising expression dynamics can reach the maximum expression level. Therefore, *BAX* and *P53DINP1* with low binding affinity [16] showed increased expression, and *CDKN1A*, *MDM2*, *GADD45* and *NOXA* with high binding affinity chose pulsing expression [9].

### p53 input frequency is optimal for driving the maximal expression of genes with decay rate that equals input frequency at the end of the 2‐th pulse

The pulsed p53 input dynamics usually consist of two pulses [10, 12]. To derive the fold change at the end of the 2‐th pulse, by inspecting Eqn (7), we can obtain the fold change at the end of the *i*‐th pulse:

$$m_{i}\left(T\right)=1+\frac{1‐\lambda^{i}}{1‐\lambda }\left(m_{1}\left(T\right)‐1\right),\lambda =e^{‐\alpha T},$$

(13) $$m_{1}\left(T\right)=1+m_{d}\left(1‐e^{‐\alpha \Delta }\right)e^{‐\alpha (T‐\Delta )}.$$

Thereafter, fold change at the end of the second pulse is

(14) $$m_{2}\left(T\right)=1+m_{d}\left(1+e^{‐\alpha T}\right)\left(1‐e^{‐\alpha \Delta }\right)e^{‐\alpha (T‐\Delta )}.$$

To find the optimal α, let $dm_{2}\left(\alpha \right)/dt=0$ yielding

$$\frac{\Delta }{T\left(1‐e^{‐\alpha \Delta }\right)}=\frac{1+2e^{‐\alpha T}}{1+e^{‐\alpha T}},$$

for the smaller α, $e^{‐\alpha \Delta }$ and $e^{‐\alpha T}$ can be replaced by 1‐αΔ and 1‐αT, respectively. As a result, we can easily obtain the optimal decay rate:

(15) $$\alpha_{2,\text{opt}}=\frac{1}{T}.$$

Thus, p53 input frequency is the optimal decay rate,

$$\alpha_{2,\text{opt}}=1/5.5\;h=0.18\;h^{‐1}.$$

The maximal fold change for target gene expression with optimal decay rate is

(16) $$m_{2,\max}=1+\frac{m_{d}\left(e^{\gamma }‐1\right)\left(e+1\right)}{e^{2}}.$$

**Fig. 2:**
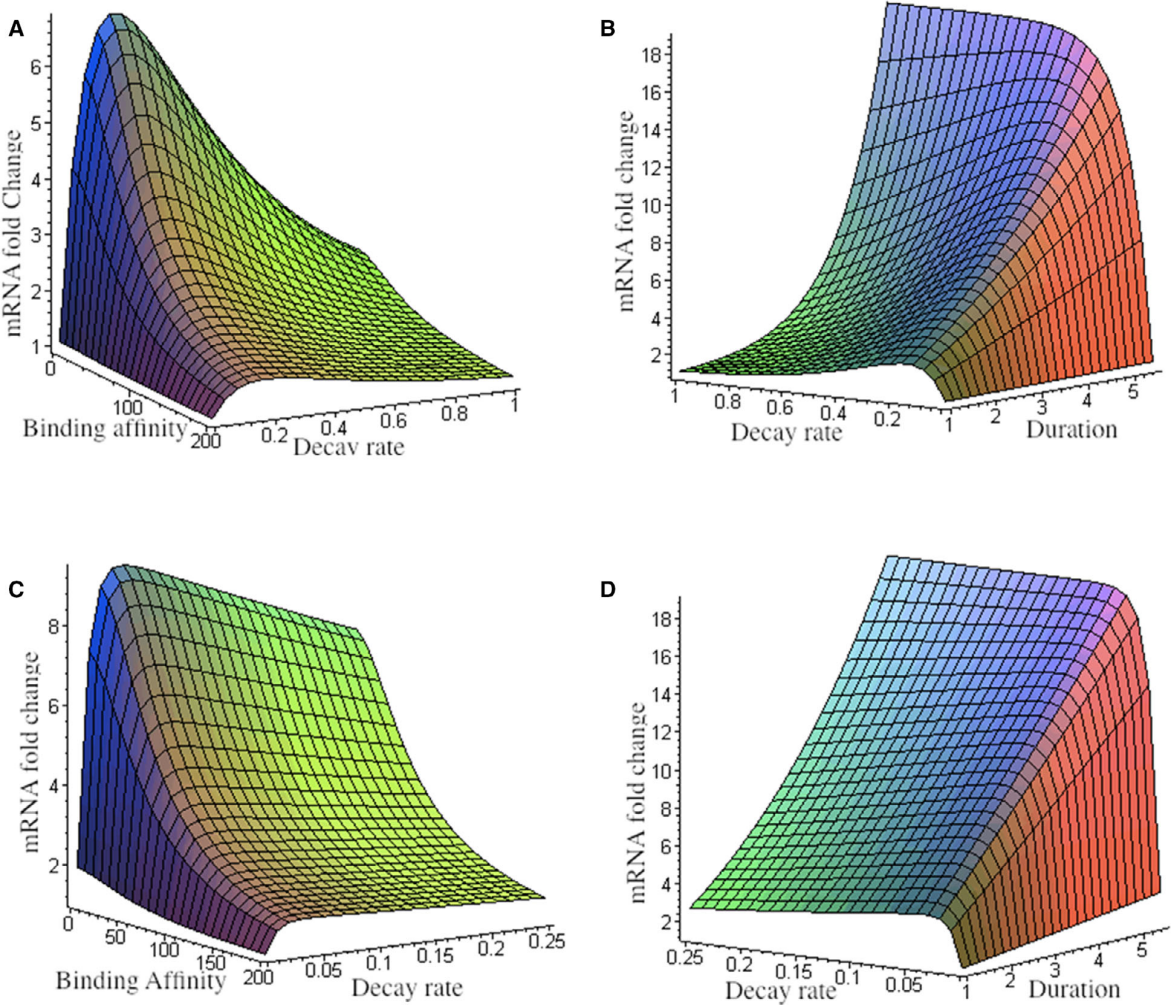
Optimal expression dynamics. (A) 2 input pulses, *T* = 5.5 h, Δ = 2.75 h, β = 18, *A* = 60 nm, *n* = 1.8. (B) 2 input pulses, *T* = 5.5 h, *K_A_* = 4.9 nm, β = 18, *A* = 60 nm, *n* = 1.8. (C) 20 input pulses, *T* = 5.5 h, Δ = 2.75 h, β = 18, *A* = 60 nm, *n* = 1.8. (D) 20 input pulses, *T* = 5.5 h, *K_A_* = 4.9 nm, β = 18, *A* = 60 nm, *n* = 1.8.

We proceeded to determine how $m_{2}\left(T\right)$ depends on certain parameters. Although the optimal decay rate is independent of binding affinity, binding affinity has a significant effect on the maximal expression level. The expression level increased sharply to a maximum when the decay rate approached the optimal value; however, at a fast decay rate, binding affinity had less effect on the expression level (Fig. 2A). Generally, the target genes with higher binding affinity have a faster mRNA decay rate, and genes with lower binding affinity have a slower decay rate. This results in an optimal equilibrium at the mRNA level. The effects of the optimal decay rate gradually become increasingly weak with an increase in duration and will eventually vanish with the duration of the pulsing period (Fig. 2B). In addition to the increase in the number of input pulses, the optimal decay rate becomes increasingly smaller, and the effects of the optimal decay rate are also weakened (Fig. 2C,D). However, the maximal expression level with 20 input pulses was slightly larger than that of two input pulses (Fig. 2A,C).

### Deciphering the number of pulses

In response to gamma irradiation, cells generally release two p53 pulses with fixed duration and 0.18 s^−1^ frequency. At the end of the 2‐th pulse, cells utilise this frequency to attain the maximal expression level in which the mRNA decay rate is the same as this frequency. We wonder if this frequency is still optimal for driving maximal gene expression under one or greater than 2 input pulses.

For one pulse input dynamics, similarly, the following is derived:

(17) $$\alpha_{\text{1,opt}}=\frac{1}{T(1‐\gamma )}>\frac{1}{T}=\alpha_{2.\text{opt}}.$$

When the number of input pulses is greater than two, the optimal decay rate is smaller than $\alpha_{2,\text{opt}}$, for example $\alpha_{20,\text{opt}}<\alpha_{2,\text{opt}}$ (Fig. 2A,C). Therefore, it is impossible for cells to select a frequency that is too high or too low. Furthermore, the mRNA decay rate of genes related to DNA repair and cell cycle arrest, such as *CDKN1A*, approaches 0.18 s^−1^; thus, under this input frequency, cells can attain the maximal expression level, stop the cell cycle, repair the damaged DNA, and avoid possible cell death from excessive expression of apoptosis genes with a smaller decay rate.

### Target gene expression with rising dynamics increases relative to the number of p53 pulses

From Eqn (7), for the smaller α, the following is obtained:

(18) $$m_{i}\left(T\right)=im_{1}\left(T\right).$$

For rising expression dynamics, mRNA fold change driven by each p53 pulse is the same; therefore, mRNA fold change after the *i*‐th pulses is *i* times that after the first pulse. In response to gamma irradiation, cells use the number of p53 pulses to encode doses of irradiation while holding duration, frequency and amplitude constant and count the number of p53 pulses to express the target gene. This counting mechanism can maintain accurate expression of target genes.

Equation (19), is useful for the expression of all target genes with rising dynamics. There are many p53 target genes with rising expression dynamics. These genes have a very small decay rate [9, 12], of which *PERP*, *S100A2*, *PYCARD*, *PIDD*, *EGFR*, *TP54i3* (*PIG3*), *DML*, *RRM2B* and *RPS27L* approach $0.01\;h^{‐1}$,while *SFN* and *DDB2* are approximately $0\text{.05}∼0\text{.1}\;h^{‐1}$. The values for the mRNA decay rate were estimated from the figure presented in the reference [9]. All genes listed above have rising expression dynamics [9, 12].

### A Hill type equation can predict the fold change of target gene expression driven by pulsing

From Eqn (7), we can obtain the average fold change over the *i*‐th period:

(19) $${\bar{m}}_{i}=\frac{1}{T}\int_{(i‐1)T}^{\mathrm{iT}}m_{i}\left(t\right)dt=1+m_{d}\left(\frac{\Delta }{T}‐\frac{\left(e^{\alpha \Delta }‐1\right)e^{‐i\alpha T}}{\alpha T}\right),$$

the stationary value is

(20) $$\bar{m}=\lim_{i\to \infty }{\bar{m}}_{i}=1+m_{d}\frac{\Delta }{T}=1+\gamma \frac{\beta A^{n}}{K_{A}^{n}+A^{n}}.$$

Equations (20 and 6) reveal that the steady state of fold change is independent of the mRNA lifetime. Under a given irradiation dose, it is assumed that the values for Δ,T,A are constant in cells, and the average β can be measured at the population level; thus, Eqn (20), can predict the fold change in cell populations. Evidently, the following equation is derived:

$$m_{\text{st,sus}}=1+m_{d}>\bar{m}.$$

From Eqn (10), we derived:

(21) $$\bar{m}=m_{\text{st,pulsed}}.$$

Thus, the observation value of the mRNA fold change at 24 h [12] can be regarded as the observation value of $\bar{m}$. Interestingly, the role of p53 pulsing is depicted only by the duty cycle. Equation (20) is of the Hill type, with six measurable parameters. Further, its conciseness allows the easy calculation of mRNA levels. The maximal fold change of each gene from the experimental data [12] can be assigned to β. The comparison of experimental measurements with theoretical predictions for some genes is shown in Table 1, where the values of parameters are from experiments, with the exception of γ, *A* and *K_A_* (*DDB2*), which are estimated. For pulsing expression, the theoretical results were higher than the experimental results. However, for increasing expression, the theoretical results were lower than the experimental results. The average error between the theoretical predictions and experiments was 23%.

**Table 1:** Theoretical and experimental fold change

Gene	Gene function	Type of dynamics	Prediction	Observation
CDKN1A	Cell cycle arrest	Pulsing	${1+2}^{3\text{.90}}\cdot 0.37\cdot \frac{60^{1.8}}{4.9^{1.8}+60^{1.8}}=6.46$	$2^{2\text{.68}}=6\text{.41}$
GADD45A	DNA repair	Pulsing	${1+2}^{3\text{.19}}\cdot 0.37\cdot \frac{60^{1.8}}{7{\text{.7}}^{1.8}+60^{1.8}}=4\text{.29}$	$2^{1\text{.584}}=3\text{.00}$
MDM2	Feedback inhibition	Pulsing	${1+2}^{2\text{.78}}\cdot 0.37\cdot \frac{60^{1.8}}{12{\text{.3}}^{1.8}+60^{1.8}}=3\text{.40}$	$2^{0\text{.999}}=2\text{.00}$
BAX	Apoptosis	Rising	${1+2}^{1\text{.23}}\cdot 0.37\cdot \frac{60^{1.8}}{73^{1.8}+60^{1.8}}=1\text{.35}$	$2^{0\text{.78}}=1\text{.72}$
DDB2	DNA repair	Rising	${1+2}^{2\text{.08}}\cdot 0.37\cdot \frac{60^{1.8}}{8^{1.8}+60^{1.8}}=2\text{.52}$	$2^{1\text{.64}}=3\text{.12}$

In the limit of very high binding affinity $K_{A}\ll A$, the Hill‐type equation can be simplified to

(22) $$\bar{m}=1+\beta \gamma .$$

For CDKN1A, which has the highest binding affinity, the expression level is easily estimated by $\bar{m}=1+2^{3.90}\cdot 0.37=6.52$, which is an approach for assessing the observation data (6.41).

### Sensitivity of the Hill type equation to parameter changes

We inspected the dependence of the Hill type equation on multiple parameters, such as duration, frequency, maximal transcription fold change and binding affinity. The relative sensitivity coefficient of $\bar{m}$ can be calculated with respect to duration Δ, frequency 1/*T*, maximal fold change β, amplitude *A* and binding affinity *K_A_* [33, 34]:

(23) $$S\left(\bar{m}‐1,\Delta \right)=\frac{\Delta }{\bar{m}‐1}\frac{d\bar{m}}{d\Delta }=1,\;S\left(\bar{m}‐1,1/T\right)=S\left(\bar{m}‐1,\beta \right)=1.$$

Similarly,

(24) $$S\left(\bar{m}‐1,A\right)=\frac{nK_{A}^{n}}{K_{A}^{n}+A^{n}}=\left\{\begin{array}{c} n{\left(\frac{K_{A}}{A}\right)}^{n},K_{A}\ll A\\ n,\;K_{A}\gg A \end{array}\right.,$$

(25) $$S\left(\bar{m}‐1,K_{A}\right)=‐\frac{nK_{A}^{n}}{K_{A}^{n}+A^{n}}=\left\{\begin{array}{c} ‐n{\left(\frac{K_{A}}{A}\right)}^{n},K_{A}\ll A\\ ‐n,\;K_{A}\gg A \end{array}\right..$$

Thus, the change in duration, frequency and maximal fold change leads to the same change in gene expression fold change. The expression levels of genes with higher binding affinity are not sensitive to changes in amplitude and binding affinity. Owing to such robustness, the p53 dynamics mainly determine the steady‐state expression level of genes with higher binding affinity, as shown in Eqn (22). In other words, the duration and frequency of p53 signalling can flexibly regulate the steady‐state expression of target genes with higher affinity. The expression level of genes with lower binding affinity is sensitive to changes in amplitude and binding affinity, and sensitivity increases with the Hill coefficient. Therefore, for *n* > 1, the cooperative binding of p53 DNA can increase the sensitivity of lower‐affinity gene expression.

### Pulsed p53 may lead to higher target gene expression than sustained p53 at low p53 levels

We sought to determine the difference in gene expression between pulsed and sustained p53 at equivalent p53 levels. The average p53 concentration is

$$\bar{p53}=\frac{1}{T}\int_{(i‐1)T}^{\mathrm{iT}}[S]dt=\gamma A.$$

Thereafter, from Eqn (21), fold change upon the pulsed condition is

(26) $$\bar{m}=m_{\text{st,pulsed}}=1+\frac{\beta_{\text{pulsed}}\gamma {\bar{p53}}^{n}}{\gamma^{n}K_{A}^{n}+{\bar{p53}}^{n}}.$$

From Eqn (6), fold change under the sustained condition is

(27) $$m_{\text{st,sus}}=1+\frac{\beta_{\text{sus}}{\bar{p53}}^{n}}{K_{A}^{n}+{\bar{p53}}^{n}}.$$

Table 1 shows that the genes with higher affinity also had higher affinity β. Because CDKN1A has the highest $\beta_{\text{pulsed}}$ and assuming that $\beta_{\text{pulsed}}=\beta_{\text{sus}}=\beta$, the equation is:

$m_{\text{st,pulsed}}‐m_{\text{st,sus}}=\frac{\beta {\bar{p53}}^{n}\left(\left(\gamma ‐\gamma^{n}\right)K_{A}^{n}‐\left(1‐\gamma \right){\bar{p53}}^{n}\right)}{\left(\gamma^{n}K_{A}^{n}+{\bar{p53}}^{n}\right)\left(K_{A}^{n}+{\bar{p53}}^{n}\right)}$.

Therefore,

(28) $$m_{\text{st,pulsed}}>m_{\text{st,sus}}\;\text{for}\;\;\bar{p53}<{\left(\frac{\gamma ‐\gamma^{n}}{1‐\gamma }\right)}^{\frac{1}{n}}K_{A}.$$

In other words, a critical condition exists where pulsed p53 can drive higher gene expression than sustained p53 at low levels of stimulation. For γ = 0.37, *n* = 1.8, the critical value is

(29) $$\bar{p53}<0.53K_{A}.$$

For CDKN1A, the condition is $\bar{\text{p53}}$ < 2.6 nm. Therefore, p53 pulses are always released from low doses of γ irradiation or nonstressed cells. In addition, p53 pulses can drive optimal gene expression to repair damaged DNA.

### mRNA half‐life determines the relaxation time of expression dynamics

The relaxation time of mRNA dynamics under pulsed signalling can be calculated using the following equation [13, 22]:

$$\tau =T\frac{\sum_{i=1}^{\infty }\bar{m}‐{\bar{m}}_{i}}{\bar{m}‐1}.$$

Thereafter, we can obtain

(30) $$\tau_{\text{pulsed}}=\frac{T(e^{\alpha \Delta }‐1)}{\alpha \Delta (e^{\alpha T}‐1)}\;.$$

The relaxation time of the mRNA dynamics driven by sustained signalling is easily obtained from Eqn (4):

(31) $$\tau_{\text{sus}}=\frac{1}{\alpha }.$$

For rising expression dynamics, when α≪1, $\tau_{\text{pulsed}}$ can be expanded in the Taylor series:

(32) $$\tau_{\text{pulsed}}\approx \frac{1}{\alpha }‐\frac{T}{2}\left(1‐\gamma \right).$$

Accordingly, the relaxation time under sustained or pulsed input is determined by the half‐life of mRNA. Of note, $\tau_{\text{sus}}>\tau_{\text{pulsed}}$ indicates that the mRNA dynamics reaches its steady state later under sustained input than under pulsed input. Compared to pulsed driving, the mRNA dynamics of p53 target genes, such as *CDKN1A*, *GADD45A*, *XPC*, *MDM2* and *PPM1D*, were delayed in the achievement of the first peaks under sustained input [8]. For example, although the dynamics of *CDKN1A* expression are weakly pulsing, the time to reach the first peak is approximately 3 h under pulsed and 7 h under sustained conditions. The expression dynamics for *BAX* and *PML* are rising expression, and the time to reach maximal level under sustained conditions is later than that under the pulsed conditions. However, the expression dynamics of genes with a smaller decay rate require a substantial time to reach the maximal level, such as *PML* (α = 0.01 h^−1^); the maximal level was observed in 12–24 h [8]. As a result, cells have enough time to repair DNA and prevent too early senescence of cells.

## Discussion

p53 dynamics control cell outcomes. The p53 downstream signalling process, such as p53 DNA binding and target gene expression, must be considered. Based on research on p53 DNA‐binding dynamics, we investigated the mRNA dynamics of p53 target genes. By using an analytical method to solve a simple model, we confirmed that there are three different types of mRNA dynamics in response to p53 input. The first type is called strongly pulsing [9] or pulses [12]; the second type, weakly pulsing [9] or induction and a plateau [12]; and the third type, rising dynamics [9] or continuous accumulation [12]. We also predicted the existence of these three patterns in p53 DNA‐binding dynamics [13].

For the third type of expression dynamics, we found that the continuous accumulation of mRNA fold change produced by each p53 pulse was the same. The number of pulses determines the total expression level and the gene that can achieve maximal induction at the end of pulses. We proved that each gene that chose this type of expression dynamics could reach the maximum expression level under a given number of input pulses. Therefore, the large number of pulses released from higher dose irradiation may drive the overexpression of BAX with lower affinity, resulting in apoptosis.

The relaxation times of the expression dynamics under pulsed or sustained p53 input were also found to be determined by the mRNA half‐life. The longer the mRNA half‐life, the longer the relaxation time. Therefore, the mRNA fold change of genes with longer half‐life, such as *BAX*, can slowly reach the maximal level, providing sufficient time for DNA repair and avoiding early apoptosis.

Fluctuations in gene expression may lead to stochasticity. Although some stochastic models include upstream periodic drives, they lack duration and binding affinity [27, 28]. By solving a deterministic model, we obtained a Hill‐type equation that can predict target gene expression driven by p53 pulses. Under conditions of high numbers of expressed mRNA, large cell volumes and fast promoter kinetics, the results from the deterministic and stochastic models are similar [29]. When the above conditions are not satisfied, the average mRNA level predicted by the deterministic model does not generally match that of the stochastic model owing to the nonlinearity in the law of mass action inherent in Eqn (1) [35].

The P53 promoter binder is similar to the drug target complex; thus, the results obtained from the p53 DNA‐binding dynamics can be applied to pulsed drug delivery [13]. Of note, the modified Hill equation rewritten in the introduction of this paper clearly shows that the longer the dissociation half‐life of the drug target complex, the better the drug efficacy. Assuming that drug target binding is cooperative (*n* > 1), thus γ < 1, pulsed drug delivery can extend the half‐life of the drug target complex. Our predictions support the lifetime of the drug target complex as the dominant factor in drug action [36].

**Table 2:** Variable and parameter definitions

Symbol	Definition	Units
*A*	p53 pulsing amplitude or sustained constant signalling	nm
*n*	Hill coefficient	–
*P*(*t*)	Function of p53 DNA‐binding dynamics	nm
mRNA(*t*)	mRNA level	nm
*m*_st_(*t*)	mRNA fold change under the sustained condition	
*m*(*t*)	mRNA fold change under the pulsed condition	–
*m_i_*(*T*)	mRNA fold change at the end of the *i*‐th pulse	
*m_d_*	Increase in steady state from sustained dynamics	
*m*_st,sus_	Steady‐state mRNA fold change under the sustained condition	–
*m*_st,pulsed_	Steady‐state mRNA fold change under the pulsed condition	–
${\bar{m}}_{i}$	Average mRNA fold change during *i*‐th pulse	–
$\bar{m}$	Stationary average mRNA fold change under the pulsed p53 input	–
*m*_0_	mRNA basal concentration	nm
α	mRNA decay rate	
α_i,opt_	Optimal mRNA decay rate under *i* input pulses	h^−1^
β	Ratio of maximal transcription rate to basal transcription rate, maximal fold change	–
β′	Maximal transcription rate	nm·h^−1^
$\beta_{0}^{\prime }$	Basal transcription rate	nm·h^−1^
*K_A_*	Dissociation constant	nm
*t*	Time	h
*T*	Period of p53 pulses	h
Δ	Duration of p53 pulses	h
γ	Duty cycle =Δ/T	‐
$\tau_{\text{sus}}$	Relaxation time to steady state under the sustained condition	h
$\tau_{\text{pulsed}}$	Relaxation time to steady state under the pulsed condition	h
$\bar{\text{p53}}$	Average p53 concentration	nm

If the maximal fold change of the pulsed input is the same as that of the sustained input, the obtained Hill‐type equation is equal to the steady state of the sustained dynamics multiplied by the duty cycle. Based on the calculations performed for the five genes, this equation agrees well with the observed results. Quantitative pharmacology was established based on the classical Hill equation [37]. Several pulsed transcription factors have been observed [4, 18]; therefore, this equation is also useful for assessing the expression dynamics of target genes driven by these transcription factors. This equation may provide cancer precision medicine with predictable results. In addition to the development of advanced optogenetic technology, precise observation of signalling molecular dynamics has become possible [38, 39]. We expect this equation to be applied extensively in the near future (Table 2).

## Author contributions

XS conceived and performed the study and wrote the article.

## Conflict of interest

The authors declare no conflict of interest'.

## Data Availability

The data that support the findings of this study are available from the corresponding author upon reasonable request.
