# Corrigendum to: A Hill type equation can predict target gene expression driven by p53 pulsing. https://febs.onlinelibrary.wiley.com/doi/full/10.1002/2211‐5463.13179

**DOI:** 10.1002/2211-5463.13838

**Published:** 2024-05-30

The above article contains an error in Eq. 18:

$$\;m_{i}\left(T\right)=im_{1}\left(T\right)$$

The corrected Eq. 18 is shown below:

$$\;m_{i}\left(T\right)=i\left(m_{1}\left(T\right)-1\right)+1$$

This change does not affect the results of the paper.

